# Establishment of patient‐derived organotypic tumor spheroid models for tumor microenvironment modeling

**DOI:** 10.1002/cam4.4114

**Published:** 2021-07-09

**Authors:** Hye Kyung Hong, Nak Hyeon Yun, Ye‐Lin Jeong, Jeehun Park, Junsang Doh, Woo Yong Lee, Yong Beom Cho

**Affiliations:** ^1^ Department of Surgery Samsung Medical Center Sungkyunkwan University School of Medicine Seoul Korea; ^2^ Institute for Future Medicine Samsung Medical Center Seoul Korea; ^3^ Department of Health Sciences and Technology SAIHST Sungkyunkwan University Seoul Korea; ^4^ Research Institute of Advanced Materials Seoul National University Seoul Korea; ^5^ Department of Materials Science and Engineering Seoul National University Seoul Korea; ^6^ Department of Biopharmaceutical Convergence Sunkyunkwan University Seoul Korea

**Keywords:** colorectal cancer, immune therapeutics, organotypic tumor spheroid, patient‐derived cancer model, tumor microenvironment

## Abstract

Patient‐derived cancer models that reconstitute the characteristics of the tumor microenvironment may facilitate efforts in precision immune‐oncology and the discovery of effective anticancer therapies. Organoids that have recently emerged as robust preclinical models typically contain tumor epithelial cells and lack the native tumor immune microenvironment. A patient‐derived organotypic tumor spheroid (PDOTS) is a novel and innovative ex vivo system that retains key features of the native tumor immune microenvironment. Here, we established and characterized a series of colorectal cancer PDOTS models for use as a preclinical platform for testing effective immunotherapy and its combinations with other drugs. Partially dissociated (> 100 μm in diameter) tumor tissues were embedded in Matrigel‐containing organoid media and subsequently formed into organoid structures within 3 to 7 days of culture. The success rate of growing PDOTS from fresh tissues was ~86%. Morphological analysis showed that the PDOTSs varied in size and structure. Immunofluorescence and flow cytometry analysis revealed that the PDOTSs retained autologous tumor‐infiltrating lymphoid cells and tumor‐infiltrating lymphoid cells were continually decreased through serial passages. Notably, PDOTSs from tumors from a high‐level microsatellite instability‐harboring patient were sensitive to anti‐PD‐1 or anti‐PD‐L1 antibodies. Our results demonstrate that the PDOTS model in which the tumor immune microenvironment is preserved may represent an advantageous ex vivo system to develop effective immune therapeutics.

## Graphical abstract

As stated in the manuscript, we established and characterized a series of colorectal cancer PDOTS models for use as a preclinical platform for testing effective immunotherapy and its combinations with other drugs. PDOTSs from tumors from a high‐level microsatellite instability‐harboring patient were sensitive to anti‐PD‐1 or anti‐PD‐L1 antibodies. Our results demonstrate that the PDOTS model in which the tumor immune microenvironment is preserved may represent an advantageous ex vivo system to develop effective immune therapeutics.

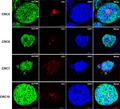

## 1 INTRODUCTION

Conventional human cancer cell lines have been widely used for preclinical studies as in vitro models to evaluate anticancer drugs. However, these cell line‐based cancer models may not reflect the characteristics of the original tissue, and most drugs that are effective in cell line‐based cancer models often fail in clinical trials.^1^ Human cancer cell lines are frequently passaged for long periods of time, which may lead to genetic alterations in these cells. In addition, almost all cancer cell lines are cultured in monolayer conditions, which are not physically representative of cancer tissues.^2^, ^3^

Patient‐derived cancer models, including primary patient‐derived cancer cells, organoids, multicellular tumor spheroids, and patient‐derived xenografts (PDXs), which recapitulate the molecular and histological features of each patient’s tumor, have been developed for various types of cancer.^4^, ^5^, ^6^, ^7^ These patient‐derived cancer models are considered the most appropriate for evaluating the efficacy of anticancer drugs and facilitating a better understanding of cancer biology. However, PDXs are expensive and labor‐intensive to generate. Organoid or multicellular tumor spheroids cultured three‐dimensionally are less time‐consuming and cheaper than PDXs, but they do not contain the native tumor microenvironment (TME).^8^, ^9^, ^10^, ^11^

There is growing interest in the study of the TME with regard to where tumor cells interact with various stromal cells, including immune cells, fibroblasts, epithelial cells, and the extracellular matrix, which play crucial roles in tumor growth and metastasis.^12^ The dynamic interactions between tumor and stromal cells in the TME impact therapy responses and are considered novel and effective therapeutic targets.^13^, ^14^, ^15^ Given the emerging importance of the TME, more appropriate cancer models that include features of the TME must be developed. In particular, immune therapeutics that accelerate anticancer immune responses through immune checkpoint inhibition have necessitated the establishment of a patient‐derived model that recapitulates the TME.^16^, ^17^, ^18^ Recently, patient‐ or murine‐derived organotypic tumor spheroids (PDOTSs/MDOTSs) that retain the native TME, including tumor‐infiltrating lymphoid and myeloid cells, have been reported as novel and innovative ex vivo systems.^19^, ^20^ Using PDOTSs/MDOTSs cultured in 3D microfluidic devices, the responses to an immune checkpoint blockade were evaluated.^19^, ^20^ In this study, we established and characterized a series of colorectal cancer PDOTS models that preserved the TME for use as a preclinical platform for testing effective immunotherapies and their combinations with other drugs.

## 2 MATERIALS AND METHODS

### 2.1 Establishment of PDOTSs

Tumor samples were collected immediately after surgery, washed with 70% ethanol and ice‐cold PBS with 3% penicillin–streptomycin (Thermo Fisher Scientific Inc., Waltham, MA, USA), and minced in a 10 cm culture dish on ice using forceps and a scalpel. The minced tumor samples were resuspended in digestion buffer [DMEM (Gibco, Thermo Fisher Scientific, Inc.) containing 1% penicillin–streptomycin (Thermo Fisher Scientific, Inc.), 2.5% FBS (Biowest, Nuaille, France), 75 U/ml collagenase type IV (Gibco, Thermo Fisher Scientific, Inc.), and 125 μg/ml Dispase II (Life Technologies, Carlsbad, CA, USA)] and incubated for 20–30 min at 37°C on a tube rotator. Following digestion, samples were pelleted and resuspended in fresh DMEM, passed through a cell strainer with a pore size of 100 μm (Falcon; BD Biosciences, NJ, USA), and centrifuged at 1000 rpm for 3 min. Then, the pellet was resuspended in DMEM and centrifuged again at 1000 rpm for 3 min to remove the debris and collagenase.

### 2.2 Culture of PDOTSs

The cell pellet was resuspended in Matrigel (BD Bioscience) on ice and plated onto 24‐well plates (50 µl of Matrigel per well). Matrigel was polymerized at 37°C for 15 min. In each well, 600–800 μl of basal culture medium [advanced DMEM/F12 supplemented with 1% penicillin/streptomycin, 10 mM HEPES, 2 mM Glutamax, 1 × B27 (all from Gibco, Thermo Fisher Scientific, Inc.), 1.25 mM N‐acetylcysteine (Sigma‐Aldrich), 100 μg/ml Primocin (InvivoGen, California San Diego, USA), and 10 mM nicotinamide (Sigma‐Aldrich)] was overlaid with the following growth factors: 1 μg/ml Human R‐spondin 1 (PeproTech, NJ, USA), 100 ng/ml Human Noggin (BioVision, CA, USA), 10 μM Y‐27632 (Selleckchem, TX, USA), 500 nM A83‐01 (Sigma‐Aldrich), 3 μM SB202190 (Sigma‐Aldrich), 50 ng/ml Human EGF (Sigma‐Aldrich), 10 nM Prostaglandin E2 (Caymanchem, MI, USA), and 10 nM Gastrin (Sigma‐Aldrich). The medium was replaced every 2 days. To passage the PDOTSs, PDOTS‐Matrigel domes were broken up by pipetting and the PDOTSs were collected in a 15 ml falcon tube. The PDOTS suspension was centrifuged at 1000 rpm for 3 min, and 1 ml of trypsin/EDTA (0.25%; Gibco; Thermo Fisher Scientific, Inc.) was added; then, the PDOTSs were incubated at 37°C for 3 min. Basal culture medium was added and the cells were spun down at 1000 rpm for 3 min. The pellet was mixed with Matrigel and 50 μl of PDOTS‐Matrigel mixture was plated in each well of the 24‐well plates. After allowing the Matrigel to solidify, basal media supplemented with growth factors were added to the plates and the PDOTSs were incubated at 37°C.

### 2.3 Hematoxylin and eosin (H&E) staining

PDOTS‐Matrigel domes were fixed with 4% paraformaldehyde for 15 min at room temperature, and the fixed domes were carefully retrieved with a spatula, placed in a mold containing optimal cutting temperature compound, and stored at −80°C. For preparing the paraffin blocks, the fixed domes were broken up by pipetting, and the PDOTSs were collected and embedded in 1% agarose. The PDOTS‐containing agarose samples were embedded in paraffin. Frozen PDOTS sections (thickness, 10 μm) were subjected to immunofluorescence staining and the paraffin‐embedded sections (thickness, 6 μm) were subjected to routine H&E staining for the examination of cell morphology.

### 2.4 Immunofluorescence staining

The frozen sections were washed with PBS, permeabilized with 0.2% Triton‐X100 in PBS for 15 min at room temperature, and incubated in blocking buffer (2% BSA and 0.2% Triton‐X100 in PBS) for 1 h at room temperature. The PDOTSs were labeled with the following antibodies: Alexa Fluor 488‐conjugated CD326 (Biolegend, San Diego, CA, USA), PE‐conjugated CD3 (BD Pharmingen), PE‐conjugated CD326 (9CA) (BioLegend), Alexa Fluor 488‐conjugated CD45 (BioLegend), Alexa Fluor 488‐conjugated CD8 (BioLegend), FITC‐conjugated CD68 (Thermo Fisher Scientific), and Alexa Fluor 488‐conjugated CD31 (BioLegend) antibodies overnight at 4–5°C in a cold room. The antibodies were diluted 1:200 in cell staining buffer (BioLegend). Coverslips were mounted onto glass slides using VECTASHIELD mounting media (Vector Laboratories) and sealed with nail polish to prevent drying, and then stored at 4°C. For live‐cell imaging, PDOTS‐Matrigel domes were grown on poly L‐lysine‐coated coverslips. After attaining 70% confluence, the PDOTS‐Matrigel domes were fixed with 4% paraformaldehyde for 1 h at room temperature, permeabilized with intracellular staining permeabilization buffer (Biolegend) for 1 h, and incubated in blocking buffer (2% BSA and 0.2% Triton‐X100 in PBS) for 1 h at room temperature. The PDOTSs were labeled with the antibodies listed above overnight at 4–5°C in a cold room. The nuclei of the PDOTSs were stained with 10 μg/ml Hoechst 33342 (Thermo Fisher Scientific). Images were obtained using a confocal laser scanning microscope (CLSM780; Carl Zeiss, Oberkochen, Germany) and the ZEN 2.3 software (Carl Zeiss).

### 2.5 Flow‐cytometric immune profiling

To analyze the tumor‐infiltrating immune cells, tumor tissues obtained by surgical operation were cut into small pieces in RPMI1640 media (Gibco) containing 3% FBS (Gibco) and 1% penicillin/streptomycin (Gibco) and were enzymatically digested using 50 U/ml collagenase A (Sigma‐Aldrich), 50 U/ml collagenase D (Sigma‐Aldrich), and 0.002% DNase (Sigma‐Aldrich) for 60 min at 37°C in a 5% CO_2_/humidified incubator. After incubation, the digested tissues were filtered through a cell strainer with a pore size of 70 μm (Corning, NY, USA) and incubated in ACK lysis buffer (Gibco) for 5 min to remove the red blood cells. Cells were stained for live/dead discrimination using a Zombie NIR fixable viability kit (BioLegend) following the manufacturer’s instructions. Prior to antibody staining, the FcR blocking reagent (Miltenyi Biotec, North Rhine‐Westphalia, Germany) was treated for 15 min at 4°C and immune cells were stained using antibodies for 20 min at 4°C. Antibodies against the following molecules: CD11b (M1/70; BUV395), CD4 (SK3; BUV 496), CD45 (HI30; BB515), CD14 (M4E2; PE), CD3 (HIT3a; APC), and HLA‐DR (G46‐6; APC‐R700) from BD Pharmingen, CD56 (5.1H11;PE) from Biolegend as well as CD8a (SK1; PerCP‐eFluor™ 710) from Invitrogen were used. The cell samples were analyzed using the FACS Aria III flow cytometer (BD Biosciences) and the data were analyzed using the FlowJo software (FlowJo LLC).

### 2.6 Live/dead imaging

Live/dead staining was performed using AO/PI Staining Solution (Nexcelom). After PD‐1 (pembrolizumab, 250 μg/ml and Nivolumab, 250 μg/ml) or PD‐L1 (atezolizumab, 600 μg/ml) treatment for 5 days, the PDOTS‐Matrigel domes were incubated with AO/PI staining solution for 20 min at room temperature in the dark. Live/dead cell images were obtained using a confocal laser scanning microscope (CLSM780; Carl Zeiss) and the ZEN 2.3 software (Carl Zeiss).

## 3 RESULTS

### 3.1 Establishment of PDOTSs from colorectal cancer tissue

**FIGURE 1:**
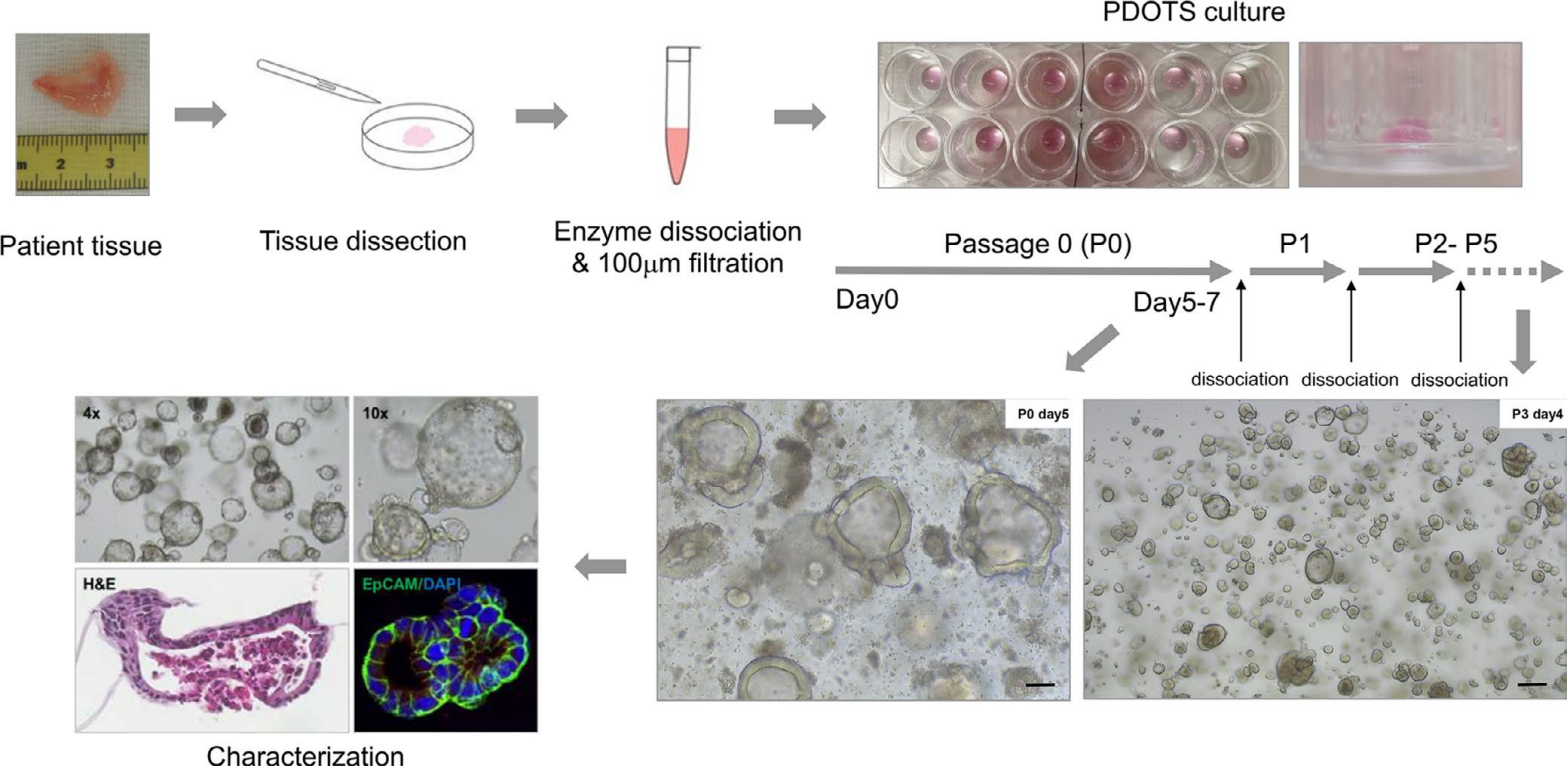
PDOTS establishment and characterization scheme. Tumor spheroids with autologous immune cells were isolated after limited collagenase digestion of fresh tumor specimens. They were embedded in Matrigel and cultured within Human organoid medium composed of Ad‐DF+++ (Advanced DMEM/F12 supplemented with 2 mM Ultra glutamine, 10 mM HEPES, and 100/100 U/ml Penicillin/Streptomycin), 100 ng/ml Noggin, 0.1 μg/ml R‐spondin 1, 1x B27 supplement, 1.25 mM N‐Acetylcysteine, 10 mM nicotinamide, 50 ng/ml human recombinant EGF, 500 nM A83‐01, 3 μM SB202190, and 10 nM prostaglandin E2. PDOTS was passaged either trituration with pipet or dissociation with trypsin for 3 min at 37°C. Scale bar, 100 μm

To establish PDOTSs, we cultured dissociated tumor tissues from 15 patients with colorectal cancer (Table 1). Following the digestion of fresh tumor samples with collagenase, organotypic tumor spheroids with autologous TMEs were isolated and cultured in Matrigel with human organoid media (Figure 1). Growth of PDOTSs was obtained with traditional cell culture media supplemented with FBS,^19^, ^20^ and we used organoid media containing growth factors to improve PDOTS growth for long‐term culture.^8^, ^9^, ^10^, ^11^ The success rate of growing PDOTS from fresh tissues was ~86%. The organotypic tumor spheroids obtained from two patients showed poor cell growth, followed by cell death over time. The PDOTSs usually formed cystic structures within 3 to 7 days of culture. When the PDOTSs grew to a size of 400 μm or became densely packed, they were passaged by either trituration with a pipette or dissociation with trypsin to establish each patient‐derived organoid (Figure 1). The established organoids were expanded for several months and cryopreserved at a low passage number (2–5 passages).

**TABLE 1:** Clinical information of patients used for PDOTS establishment

Patient ID	Age	Sex	Tumor	Cell type	MSI status	Stage	PDOTS
CRC1	63	F	Ascending colon	WD	MSS	1	O
CRC2	54	F	Hepatic flexure colon	MD	MSS	1	O
CRC3	76	M	Rectosigmoid junction	Mucinous	MSS	3	X
CRC4	74	F	Sigmoid colon	Mucinous	MSI‐H	3	O
CRC5	53	M	Rectum	WD	MSS	3	O
CRC6	60	M	Ascending colon	PD	MSI‐H	2	O
CRC7	28	F	Rectosigmoid junction	MD	MSS	3	O
CRC8	53	F	Ascending colon	MD	MSS	2	O
CRC9	49	M	Sigmoid colon	WD	MSS	1	X
CRC10	61	M	Sigmoid colon	WD	MSS	2	O
CRC11	79	F	Ascending colon	WD	MSS	3	O
CRC12	53	F	Rectosigmoid junction	MD	MSS	3	O
CRC13	54	M	Upper rectum	MD	MSI‐H	2	O
CRC14	68	M	Ascending colon	MD	MSI‐L	3	O
CRC15	51	M	Splenic flexure colon	Mucinous	MSS	2	O

Abbreviations: MD, moderately differentiated; MSI‐H, microsatellite instability‐high; MSI‐L, microsatellite instability‐low; MSS, microsatellite stable; O, success to establish organoids; PD, poorly differentiated; X, fail to establish organoids; WD, well differentiated.

### 3.2 Characterization of the PDOTSs

**FIGURE 2:**
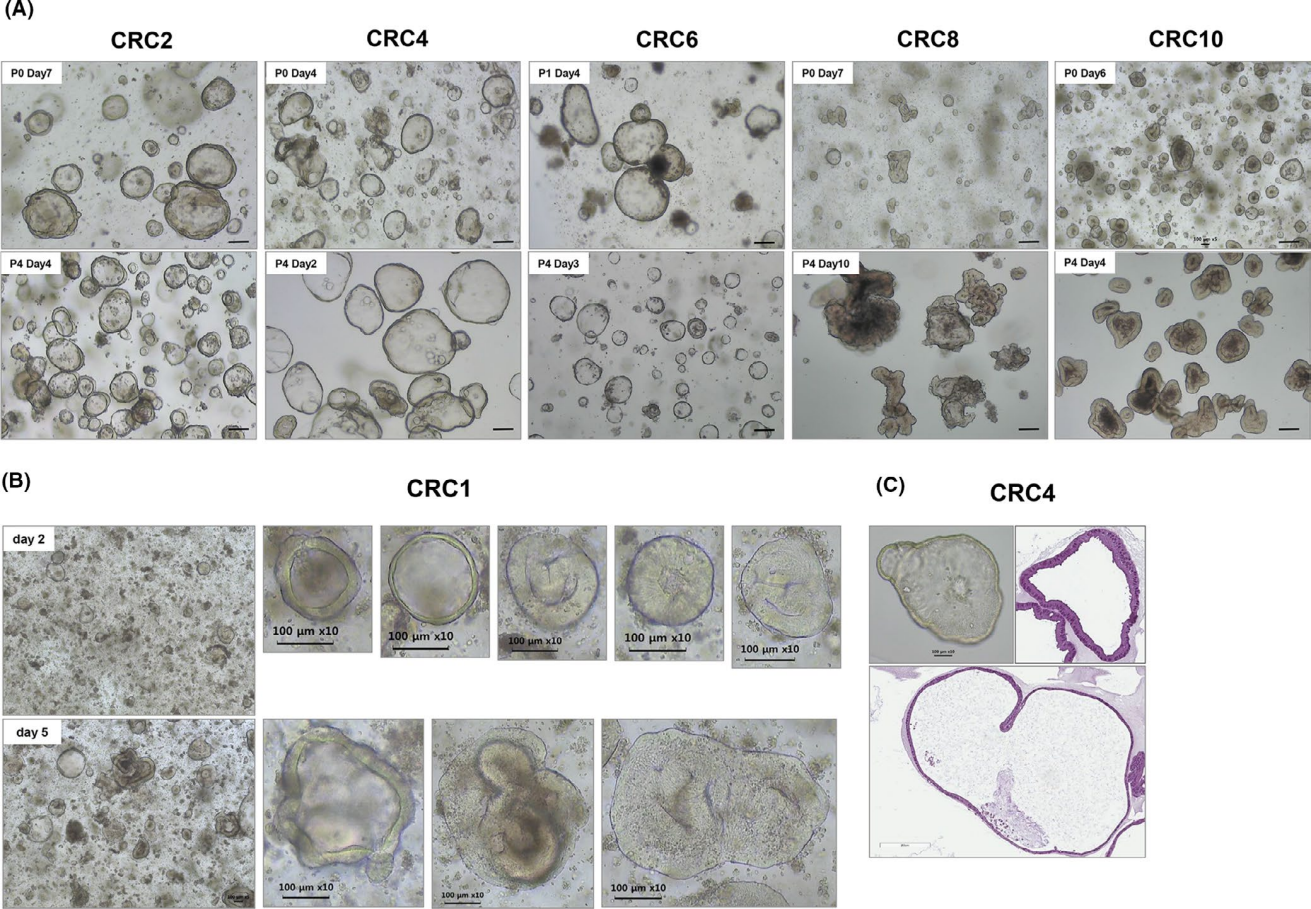
PDOTS morphology in bright‐field microscopy. Multicellular OTS derived from colorectal cancer patients were observed in the phase‐contrast imaging. (A) PDOTS morphology remained the same cystic and/or luminal structures in both Passage 0 and Passage 4. (B) The size of the CRC1 PDOTS was approximately 100 μm following 2 days of culture; they increased in size by twofold within 5 days (C) H & E staining of OTS from CRC4 patient tumor tissue. Scale bar, 100 μm

Morphological analysis showed that the PDOTSs varied in size and structure between patient samples, ranging from thin‐walled cystic structures to compact organoids devoid of a lumen (Figure 2A). The structure and morphology of the PDOTSs were preserved through several passages. Some PDOTS continued to grow beyond 7 days and formed large thin‐walled cystic structures (> 400 μm in size) with lumens. The initial size of the PDOTSs was approximately 100 μm following 2 days of culture; they increased in size by twofold within 5 days (Figure 2B). H&E staining confirmed that most PDOTS had a well‐differentiated epithelial layer with a luminal area (Figure 2C).

### 3.3 The PDOTSs preserved the immune elements of the TME

**FIGURE 3:**
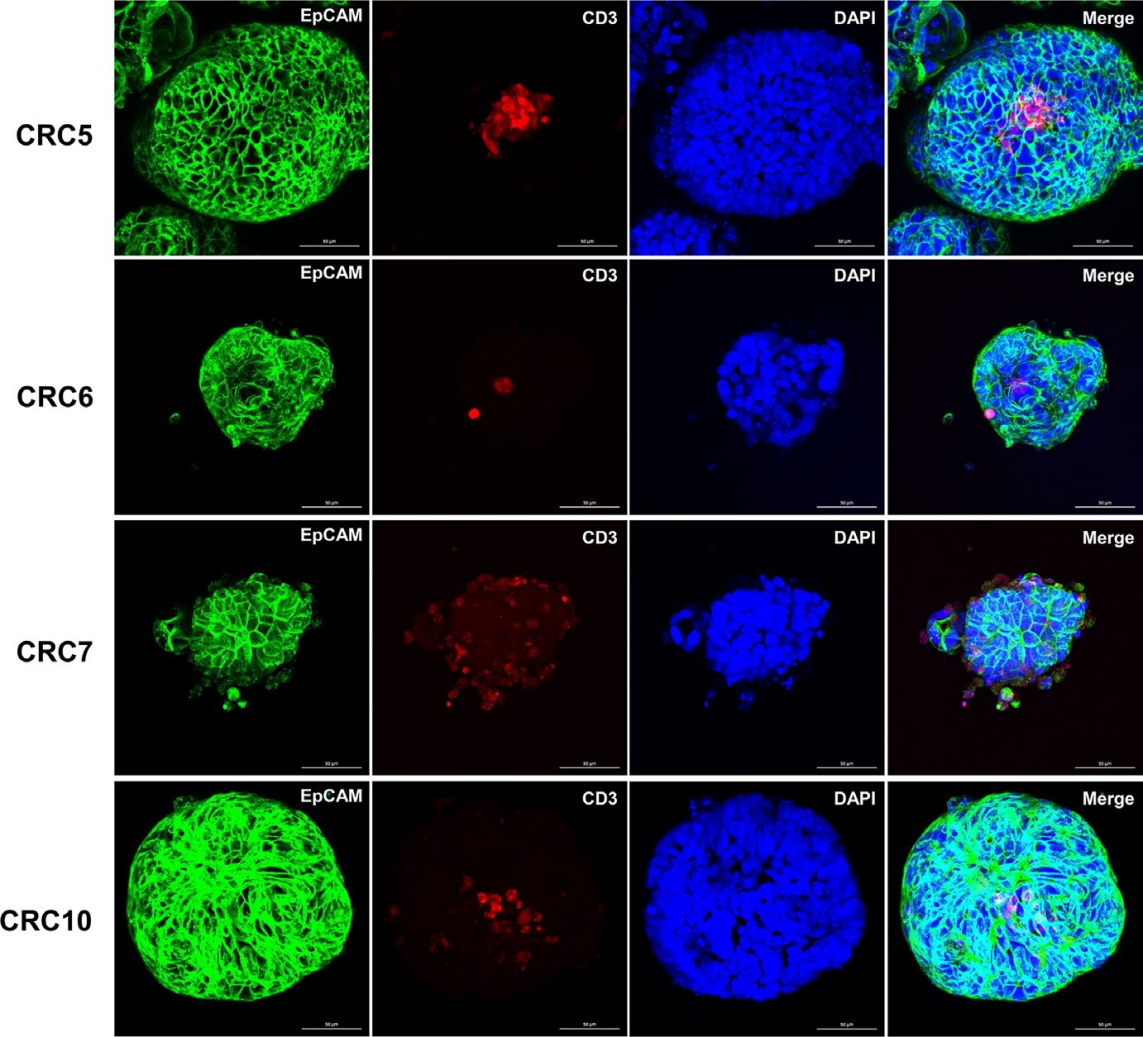
Immunofluorescent Characterization of PDOTSs. PDOTS contained CD3+ T cells integrally embedded in close proximity to tumor cells. Tumor cells were readily detected using EpCAM antibody (green). CD3+ T cells were readily detected using CD3 antibody (red). DAPI was used for nuclear staining (Blue). First panel figures (CRC5) were adapted from reference 6. Scale bar, 100 μm

**FIGURE 4:**
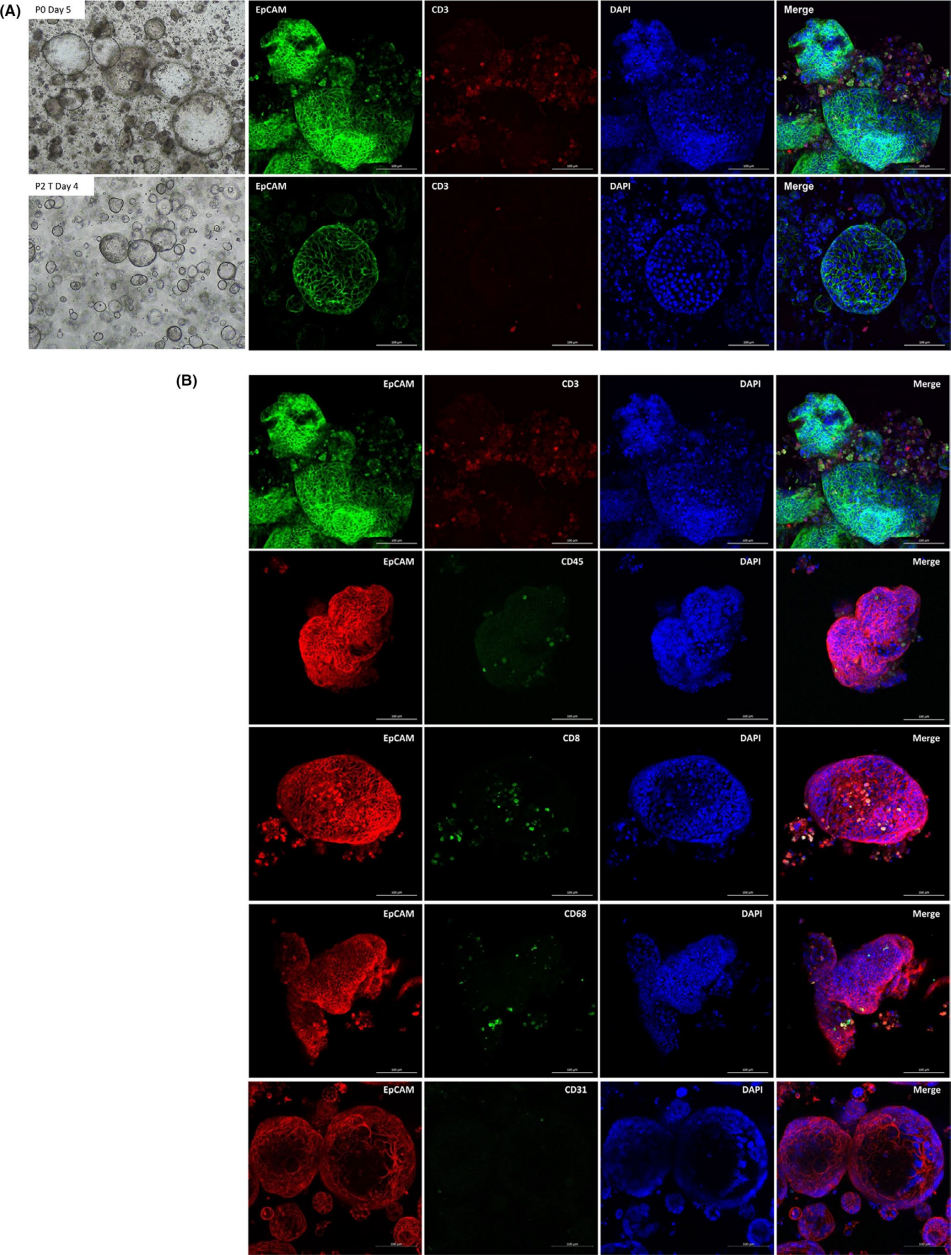
PDOTS preserve diverse stromal elements. (A) CD3+ T‐cell preservation in Passage 0 (P0) PDOTS culture day 5 and in Passage 2 (P2) PDOTS culture day 4. Green, EpCAM+ epithelial cells; Red, CD3+ T cells; Blue, DAPI. (B) Various levels of stromal elements in Passage 0 (P0) PDOTS culture day 5. First panel figures were adapted from (A) upper panel. Red, EpCAM+ epithelial cells; Green, CD45+ T cells; Green, CD8+ T cells, Green, CD68+ macrophages, and Green, CD31+ endothelial cells; Blue, DAPI. Scale bar, 100 μm

**FIGURE 5:**
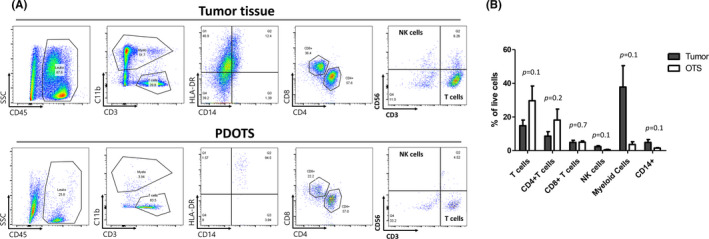
Immune cell profiling of PDOTS. (A) Representative dot plot images of immune cell analysis. Singlets, live cells, and whole immune cells (leukocytes) were sequentially gated. Leukocytes were divided to myeloid cells and T‐cell population. Myeloid cells were further analyzed using CD14 and HLA‐DR. T cells were further analyzed to CD4+ T cells and CD8+ T cells. NK cells were analyzed using CD56. (B) Flow cytometry analysis of immune cell population in tumor and OTS. Mean and SEM were plotted

The initial culture of the PDOTSs included a mixed population of epithelial tumor cells and various stromal cells. We investigated the presence of immune cells from the PDOTSs using confocal microscopy. The PDOTSs contained tumor‐infiltrated CD3+ T cells that were embedded in the luminal area or in close proximity to tumor epithelial cells (Figures 3). These CD3+ T cells existed in the PDOTSs but their numbers decreased after serial passages (Figure 4A). The PDOTSs also contained various levels of CD45+, CD8+, CD68+ macrophages, and CD31+ endothelial cells (Figure 4B). To compare the immune cell populations between tumor tissues and the PDOTSs, T cells, CD4+, CD8+, myeloid cells, especially those of the monocyte lineage (CD14+), and NK cells (CD56+) were analyzed by flow cytometry (Figure 5A). The PDOTSs showed a smaller myeloid cell population, including monocyte lineage CD14+ cells, than the tumor tissues due to the death of myeloid cells in the PDOTSs. The relative T‐cell ratio was higher in the PDOTSs than in the tumor tissues, and this was caused by the decreased numbers of live myeloid cells in the PDOTSs. In the T‐cell subpopulation, a higher number of CD4+ T cells than CD8+ cells were maintained in both the PDOTSs and tumor tissues. NK cells existed in tumor and OTS but they were relatively minor immune cell population than T cells (Figure 5B).

### 3.4 Sensitivity of the PDOTSs toward immune checkpoint blockades

**FIGURE 6:**
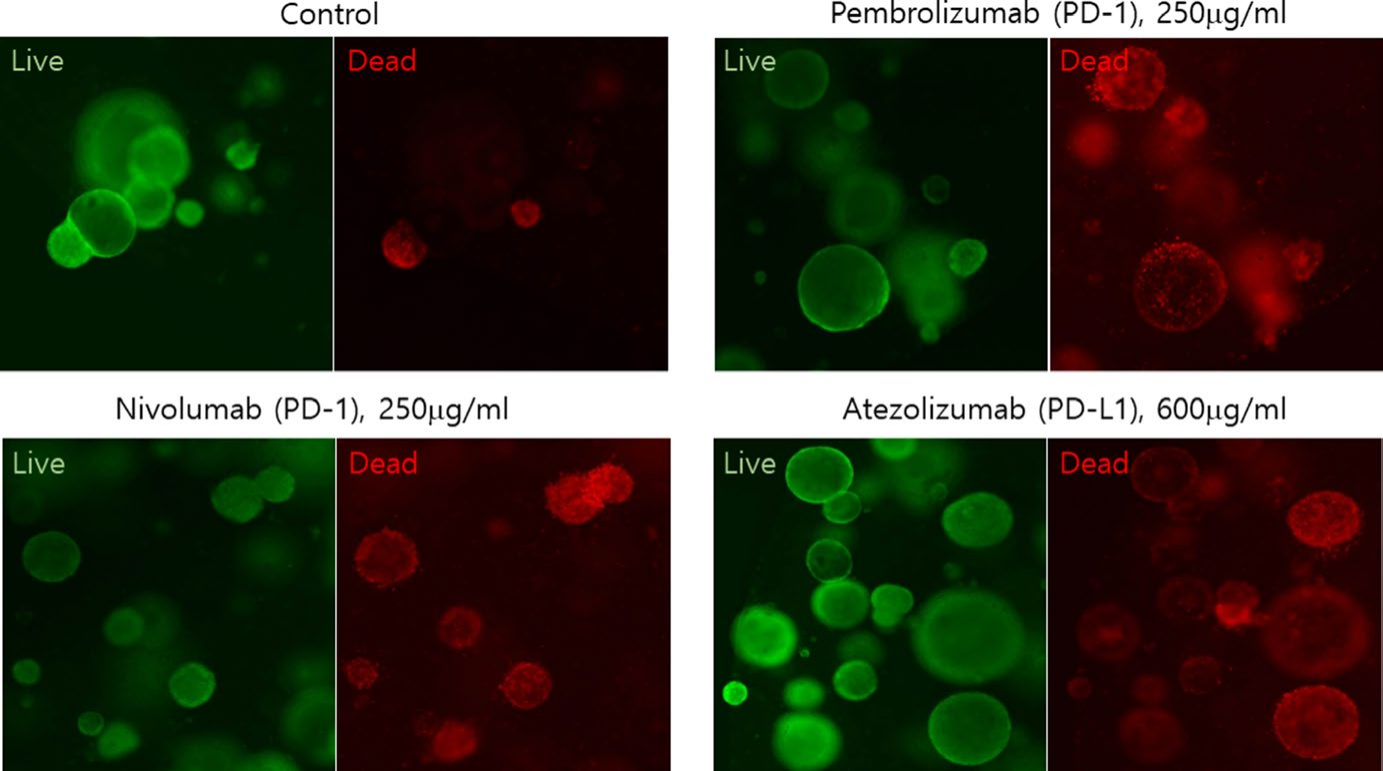
The response of PDOTSs to immune checkpoint blockades. Live/dead analysis of PDOTS performed on day 5 following treatment with PD‐1 (pembrolizumab, 250 μg/ml and Nivolumab, 250 μg/ml) or PD‐L1 (atezolizumab, 600 μg/ml, *n* = 3, biological replicates; representative images shown). Scale bar, 200 μm

To demonstrate the response of PDOTSs to immune checkpoint blockades, PDOTSs from a tumor from a high‐level microsatellite instability (MSI‐H)‐harboring patient (CRC13) were treated with anti‐PD‐1 or anti‐PD‐L1 antibodies for 7 days. Acridine orange (live cells) and propidium iodide (dead cells) labeling were used for live/dead cell staining of the PDOTSs after drug treatment. The PDOTSs with MSI‐H were sensitive to anti‐PD‐1 and anti‐PD‐L1 antibodies (Figure 6). Flow cytometric analysis revealed larger CD4+ and CD8+ T‐cell populations in the PDOTSs (CRC13); these cells are necessary effectors following treatment with anti‐PD‐L1 antibodies (Figure 5A).

## 4 DISCUSSION

The development of a human cancer model for predicting the anticancer drug sensitivity of individual patients is needed to improve the clinical outcomes. Patient‐derived cancer models, which include organoids, multicellular tumor spheroids, and PDXs, are considered to reflect the original tumors more closely than conventional cancer models and are used to screen for novel anticancer drugs.^5^, ^21^, ^22^, ^23^, ^24^ However, the establishment of PDXs is time‐consuming, expensive, and associated with a low‐throughput. Organoids or other tumor spheroids do not contain the TME, which crucially affects drug responses.^8^, ^9^, ^10^, ^11^

Immunotherapy is a major paradigm shift in current cancer drug development and cancer biology. In particular, immune checkpoint blockades targeting PD‐1, PD‐L1, and CTLA4 have demonstrated promising clinical activity against several tumor types.^25^ Although current immunotherapies show impressive clinical responses in melanomas and other cancers, the majority of patients with cancer do not respond to these therapeutics.^26^ Currently, there is no appropriate model available that represents the interactions of tumor and immune cells in the TME, and it is currently unclear which patients will respond to immunotherapies.^27^ Recently, Jenkins et al ^19^ demonstrated that PDOTSs and MDOTSs contain relatively similar subsets of cells from lymphoid and myeloid populations that grow readily in collagen hydrogels in a 3D microfluidic culture system.^19^, ^20^ Using these models, the authors evaluated the response to the PD‐1/PD‐L1 blockade and recapitulated the features of ex vivo sensitivity and resistance to the PD‐1 blockade.

In this study, we established a series of PDOTSs from partially dissociated (> 100 μm in diameter) tumors of patients with colorectal cancer. Characterization of the PDOTSs showed that autologous tumor‐infiltrating T lymphocytes (CD3+, CD4+, and CD8+) were retained during short‐term in vitro culture. PDOTSs do not require days or weeks of culture. Despite concerns that organoid media would be unsuitable for immune cell viability, our study shows that relevant tumor‐infiltrating CD3+, CD4+, and CD8+ cells can be successfully retained in organoid media. Organoids have been rapidly adapted to patient‐derived cancer modeling.^28^ Early patient‐derived organoid models of colorectal cancer comprised only tumor epithelial cells without stromal components.^5^, ^11^, ^29^, ^30^ Recently, various organoid culture methods in which cancer cells are grown with TME‐associated immune components have been reported.^28^ Epithelial tumor‐only organoids can be reconstituted with exogenously added immune components from autologous peripheral blood or tumor tissues.^31^ In addition, the air–liquid interface method can recapitulate the native TME by preserving diverse endogenous immune cells without reconstitution.^32^ These models have been applied to make them more suitable for evaluating the response to immunotherapies and for assessing novel therapeutics.

Our results demonstrate the potential of PDOTSs as a patient‐derived cancer model for the predictive assessment of individualized patient responses to current immunotherapies. Additional studies are required to extend the long‐term preservation of other cell types, such as fibroblasts, endothelial cells, and other immune cells that are located in the TME. In addition, future studies will be conducted to establish definitive correlations between PDOTSs and immunotherapy responses by analogy to 3D microfluidic approaches. Three‐dimensional microfluidic culture of PDOTSs may provide a more realistic environment in which tumor‐TME interactions could be evaluated; they could be used as an ex vivo system to drive translational efforts to develop personalized immunotherapies.

## 5 ETHICS APPROVAL STATEMENT

Patient samples were obtained from the Department of Surgery at the Samsung Medical Center in accordance with protocols approved by the Institutional Review Board (IRB approval no. SMC2019‐11‐129‐001). Surgical tumor specimens and clinical records were obtained from patients with informed written consent.

## CONFLICT OF INTEREST

The authors declare no conflict of interest.

## Author contributions

Yong Beom Cho conceived of the study and participated in its design. Hye Kyung Hong and Nak Hyeon Yun participated in the design of the study, interpreted the data, and drafted the manuscript. Hye Kyung Hong, Ye‐Lin Jeong, and Woo Yong Lee carried out the patient‐derived organotypic tumor spheroid establishment and characteristics including histological analysis, immunofluorescence staining, and live/dead imaging and the study coordination. Jeehun Park and Junsang Doh carried out the flow‐cytometric immune profiling analysis and interpreted the data and helped to draft the manuscript. All authors read and approved the final manuscript.

## Data Availability

The data that support the findings of this study are available from the corresponding author upon reasonable request.
